# Gastrectomy with or without Complete Omentectomy for Advanced Gastric Cancer: A Meta-Analysis

**DOI:** 10.3390/medicina58091241

**Published:** 2022-09-07

**Authors:** Maurizio Zizzo, Magda Zanelli, Francesca Sanguedolce, Andrea Palicelli, Stefano Ascani, Andrea Morini, David Tumiati, Federica Mereu, Antonia Lavinia Zuliani, Melissa Nardecchia, Francesca Gatto, Manuel Zanni, Alessandro Giunta

**Affiliations:** 1 Surgical Oncology Unit, Azienda Unità Sanitaria Locale-IRCCS di Reggio Emilia, 42123 Reggio Emilia, Italy; 2 Pathology Unit, Azienda Unità Sanitaria Locale-IRCCS di Reggio Emilia, 42123 Reggio Emilia, Italy; 3 Pathology Unit, Azienda Ospedaliero-Universitaria, Ospedali Riuniti di Foggia, 71122 Foggia, Italy; 4 Hematology Unit, CREO, Azienda Ospedaliera di Perugia, University of Perugia, 06129 Perugia, Italy; 5 Pathology Unit, Azienda Ospedaliera S. Maria di Terni, University of Perugia, 05100 Terni, Italy

**Keywords:** omentum, omentectomy, gastric cancer, gastrectomy, outcomes

## Abstract

*Background and Objectives*: Surgery remains the only possible curative treatment for advanced gastric cancer (AGC). Peritoneal metastases are estimated to occur in approximately 55–60% AGC patients. Greater omentum is the most common metastatic area in AGC. At present, omentectomy alone or bursectomy are usually carried out during gastric cancer surgery. We performed a meta-analysis in order to evaluate long-term and short-term outcomes among AGC patients, who have undergone radical gastrectomy with or without complete omentectomy (CO). *Materials and Methods*: We performed a systematic review following the Preferred Reporting Items for Systematic Reviews and Meta-Analyses (PRISMA) guidelines. Meta-analysis was performed by use of RevMan (Computer program) Version 5.4. *Results*: The eight included studies covered an approximately 20 years long study period (2000–2018). Almost all included studies were retrospective ones and originated from Asian countries. Meta-analysis indicated gastrectomy without CO as significantly associated with longer 3-year (RR: 0.94, 95% CI: 0.90–0.98, *p* = 0.005) and 5-year overall survivals (OS) (RR: 0.93, 95% CI: 0.88–0.98, *p* = 0.007). Moreover, we found longer operative time (MD: 24.00, 95% CI: −0.45–48.45, *p* = 0.05) and higher estimated blood loss (MD: 194.76, 95% CI: 96.40–293.13, *p* = 0.0001) in CO group. *Conclusions*: Non-complete omentectomy (NCO) group had a statistically greater rate in 3-year and 5-year OSs than the CO group, while the CO group had significantly longer operative time and higher estimated blood loss than the NCO group. Further randomized, possibly multi-center trials may turn out of paramount importance in confirming our results.

## 1. Introduction

According to GLOBOCAN 2020 data, gastric cancer (GC) is the fifth most frequent malignancy and the third most common cause of cancer-related death worldwide [1,2]. Gastrectomy with D2 lymphadenectomy remains the only possible curative treatment for advanced gastric cancer (AGC), in spite of recent and ongoing developments in neoadjuvant/adjuvant chemotherapy as well as molecular-targeted agents [3,4].

Although many therapeutic advancements have been achieved in recent decades, GC remains an easily progressing, recurring and metastasizing condition marked by a <35% 5-year overall survival (OS) rate [5].

Being the most common recurrence in GC, peritoneal metastases are estimated to occur in approximately 55–60% AGC patients, thus being deemed a leading factor in GC poor outcomes [5]. Peritoneal recurrence is commonly detected in patients undergoing gastrectomy for cT3 and cT4 GC [6,7]. As concerns metastatic peritoneal sites, the greater omentum is the most common metastatic area in AGC. At present, omentectomy (resection of the greater omentum) alone or bursectomy (resection of the bursa omentalis: greater omentum, anterior membrane of transverse mesocolon and pancreatic capsule) are usually carried out during GC surgery [8,9,10,11,12]. However, bursectomy did not provide a survival advantage over non-bursectomy (omentectomy alone) in patients with resectable AGC, as demonstrated by relevant results from the JCOG1001 trial [13]. In particular, the authors concluded that D2 lymphadenectomy with omentectomy alone should be performed as standard surgery for resectable cT3-T4a GC [13].

From a purely anatomical perspective, resection of the bursa omentalis or omentum alone should be an integral part of radical resection for AGC patients [14]. Both areas could represent preferential implantation sites of GC metastases, due to the presence of omental “milky spots”, i.e., clusters of immune cells in the omentum, that are supposed to play a paramount role in determining both transit and anchoring of cancer cells [8,9,10,11,12,14,15].

Several mainly retrospective observational studies analyzed the role of complete omentectomy (CO) in addition to radical gastrectomy in GC patients [16,17,18,19,20,21]. Given the presence of significant bias in the aforementioned studies, different meta-analyses have been developed in order to improve the present evidence [6,7,14,22,23,24,25,26]. Such meta-analyses demonstrated that gastrectomy without CO gives similar, even better short- and long-term results than gastrectomy with CO, although many of those studies also included populations with early gastric cancer (EGC) [6,7,14,22,23,24,25,26]. At present, outcomes of just one randomized controlled trial (RCT) are available [27], while two other RCTs are being investigated [28,29].

Following a recent non-systematic literature search, we identified another study that compared gastrectomy without CO with gastrectomy with CO for AGC [30]. Therefore, our meta-analysis aimed at providing an update on current evidence coming from a comparison between long-term and short-term outcomes among AGC patients who have undergone radical gastrectomy with or without CO.

## 2. Materials and Methods

The present meta-analysis was performed following the Preferred Reporting Items for Systematic Reviews and Meta-Analyses (PRISMA) statement and guidelines [31]. Since this meta-analysis was based on previously published studies and no original patient population data have been added, ethics committee approval and informed patient consent are not required.

Before the start of the search, a review protocol was entered into the PROSPERO database (CRD42022339519).

### 2.1. Search Strategy

PubMed/MEDLINE, Scopus, Cochrane Library (Cochrane Database of Systematic Reviews, Cochrane Central Register of Controlled Trials-CENTRAL) and Web of Science (Science and Social Science Citation Index) databases were used to identify articles of interest.

Combination of non-MeSH/MeSH terms was as follows:

- PubMed/MEDLINE

((omentectomy[Title/Abstract]) AND (gastrectomy[Title/Abstract])) AND (gastric cancer[Title/Abstract]) Filters: English.

- Scopus

(TITLE-ABS-KEY (omentectomy) AND TITLE-ABS-KEY (gastrectomy) AND TITLE-ABS-KEY (gastric AND cancer)) AND (LIMIT-TO (LANGUAGE, “English”)).

- Cochrane Library

omentectomy in Title Abstract Keyword AND gastrectomy in Title Abstract Keyword AND gastric cancer in Title Abstract Keyword—(Word variations have been searched).

- Web of Science

((Topic = (omentectomy)) AND Topic = (gastrectomy)) AND Topic = (gastric cancer).

Refined by: LANGUAGES: ENGLISH.

Final analysis was carried out in 3 June 2022.

Moreover, the reference lists of included studies and relevant reviews were manually searched.

### 2.2. Inclusion Criteria

Comparative population studies (case series, case-control studies, cohort studies, controlled clinical trials and randomized clinical trials) were included that involved adult patients (over 18 years of age) undergoing radical gastrectomy with or without CO for pathologically confirmed AGC. Abstracts, posters, letters to the Editor, editorials, case reports and previously published systematic reviews and/or meta-analyses were ruled out.

Furthermore, studies comparing CO with non-complete omentectomy (NCO) in patient populations having just EGC or mixed ECG + AGC data were ruled out, as well as studies involving mixed populations undergoing gastrectomy with or without CO for mixed benign and malignant gastric diseases.

Only scientific papers in the English language were included.

### 2.3. Outcomes

Primary outcomes included 3-year and 5-year overall survival rates, 3-year and 5-year disease-free survival (DFS) rates, overall and specific recurrence (peritoneal and other sites) of each eligible trial, while secondary outcomes included assessment of short-term ones, as operative time, estimated blood loss, number of harvested lymph nodes and postoperative data such as overall complications and major complications.

### 2.4. Data Extraction

Papers were selected and identified by two independent reviewers (Maurizio Zizzo and A.M.) based on title, abstracts, keywords and full texts. Following data were collected from included papers:

- Demographic data [Author’s surname and year of publication, study type, study country, population size, gender and age, body mass index (BMI)];
- Surgical data [surgical approach, surgical procedure];
- Histopathological data [primary GC T stage, lymphadenectomy extension];
- Outcomes data [3-year and 5-year OSs, 3-year and 5-year DFSs, overall and specific recurrence rates, harvested lymph nodes number, overall and major complications].

All collected results were eventually reviewed by a third independent reviewer (A.G.).

### 2.5. Quality Assessment

For a proper quality assessment of the different included studies, two independent reviewers analyzed the risks of biased assessments (RoB 2 and ROBINS-I) [32,33].

Version 2 Cochrane Risk-of-Bias tool for randomized trials (RoB 2) was recommended in assessing the risk of bias in randomized trials [32]. It included a fixed set of bias domains that were focused on different aspects of study design, conduct and reporting [32]. Within each domain, a series of questions (“reporting questions”) aimed at collecting data on study features that were relevant to the risk of bias [32]. A proposal for bias risk from each domain was generated by an algorithm, based on answers to reporting questions [31]. Ratings for risk of bias were “Low”, “High”, or “Some Concerns” [32].

ROBINS-I tool was developed to assess the risk of bias in non-randomized studies, that compared health outcomes of two or more interventions [33]. In order to obtain an assessment of the risk, reporting questions were used that had a substantial factual nature and aimed at easing judgment on the risk of bias [33]. Answers to the reporting questions provided a framework for domain-level judgments on the risk of bias, which then served as a basis for an overall judgment on the risk of bias in a special outcome [33]. Ratings for risk of bias judgments were “Low Risk”, “Moderate Risk”, “Severe Risk” and “Critical Risk”, keeping in mind that “Low risk” meant the risk of bias in a high-quality randomized study [33]. Only in exceptional cases will a non-randomized study be rated as low risk of bias due to confounding variables [33].

### 2.6. Statistical Analysis

Our meta-analysis was performed by use of “Review Manager (RevMan) [Computer program] Version 5.4. The Cochrane Collaboration, 2020” [34]. In the case of dichotomous outcomes, risk ratios (RRs) and the corresponding 95% confidence intervals (CIs) were computed according to Mantel–Haenszel (MH) method. For continuous outcomes, weighted mean differences (WMD) and corresponding 95% CI were calculated by use of the inverse variance method. In the lack of mean or standard deviation (SD) of an endpoint, it was calculated from the reported median range, or interquartile range (IQR), if provided.

I^2^ statistics were used to assess the statistical heterogeneity. <25, 25–50 and >50% I^2^ values were classified as low, moderate and high heterogeneity, respectively. Due to heterogeneity of malignant disease and patient features, in addition to discrepancies in surgical approaches and adopted methods, a random-effects model was used as the default for all statistical analyses. Statistical significance was set at *p* < 0.05. Moreover, subgroup analysis stratified by different study designs was performed.

## 3. Results

### 3.1. Search Results and Study Characteristics

**Figure 1:**
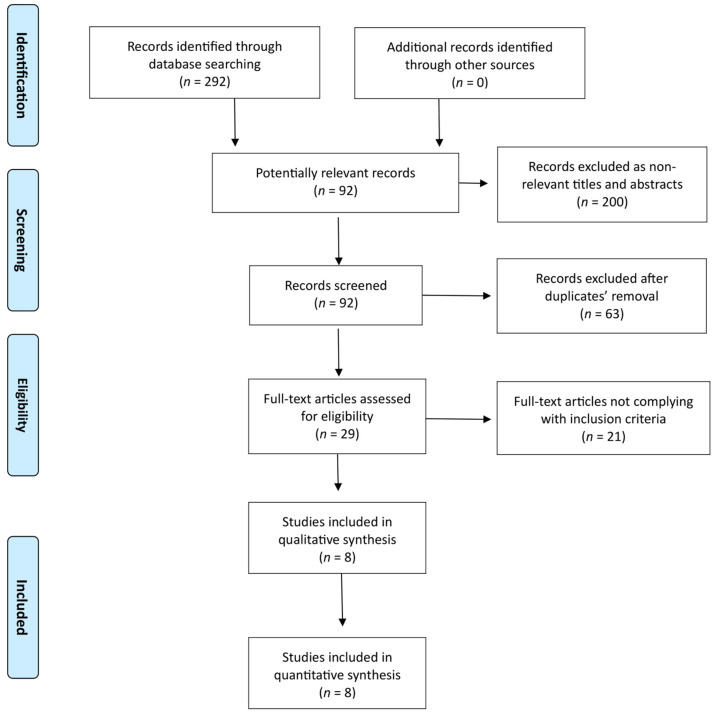
PRISMA flow chart of literature search.

According to the final literature search, which was performed on 3 June 2022, 292 potentially interesting studies were found (PubMed/MEDLINE: 53 records; Scopus: 148 records; Cochrane Library: 25 records; Web of Science: 66 records) (Figure 1). Although analysis covered all 292 studies, 200 ones turned out as not relevant for title and abstract, while 92 full-texts were considered eligible. Then, 63 out of 92 studies were ruled out as duplicate publications. Following the exclusion of 21 studies not complying with inclusion criteria, 8 comparative studies (seven retrospective studies and one randomized controlled study) underwent qualitative and quantitative synthesis [16,17,18,19,20,21,27,30]. No additional record was found through other sources (References list). Six out of the seven retrospective studies used propensity score matching (PSM) analysis [16,17,19,20,21,30].

According to ROBINS-I, most non-randomized studies showed moderate overall bias, except for Young et al.’s (serious) (see Supplementary Materials, Table S1). On the contrary, according to RoB 2, the only included randomized study had a low overall bias (see Supplementary Materials, Table S2).

### 3.2. Study and General Population Characteristics

Table 1 and Table 2 show study characteristics and demographic features of analyzed populations. The identified eight studies covered an almost 20-year period (2000–2018) [16,17,18,19,20,21,27,30]. In total, 43.2% of the general population (1122/2598) underwent gastrectomy and CO [16,17,18,19,20,21,27,30]. We recorded a male preponderance (66.4%; 1725/2598) and a 56–74-year-old population [16,17,18,19,20,21,27,30].

Moreover, 54.1% (1150/2127) and 64.5% (1372/2127) patients underwent open approach and distal gastrectomies, respectively [16,17,18,19,20,21,27,30]. Most gastrectomies were of the D2/D2+ type (75.8%; 1292/1705) [16,17,18,19,20,21,27,30]. pTNM stages III/IV were slightly predominant (51.2%; 1018/1987) [16,17,18,19,20,21,27,30].

### 3.3. Meta-Analyses Results

#### 3.3.1. Overall Survival

**Figure 2:**
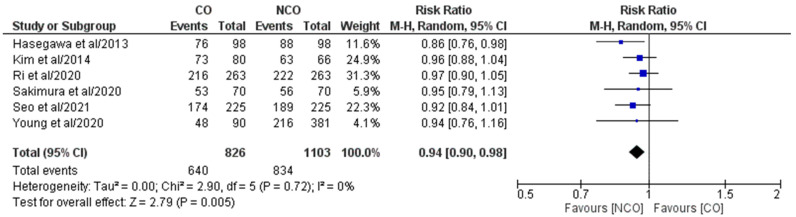
Forest plot comparing 3-year OS between the CO and NCO groups. CI, confidence interval; M–H, Mantel–Haenszel [16,17,18,19,20,21].

**Figure 3:**
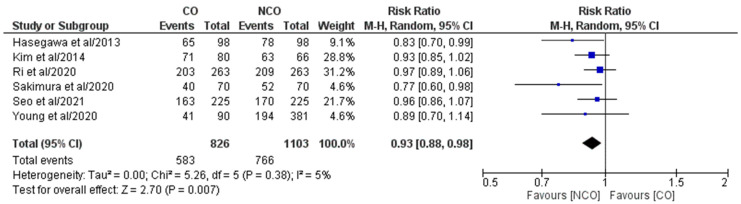
Forest plot comparing 5-year OS between the CO and NCO groups. CI, confidence interval; M–H, Mantel–Haenszel [16,17,18,19,20,21].

Overall, six studies out of the eight included ones [16,17,18,19,20,21] comprising 1929 patients (CO 826, NCO 1103) who reported both 3-year and 5-year Oss (Figure 2 and Figure 3). The meta-analysis of pooled results showed that 3-year OS (RR: 0.94, 95% CI: 0.90–0.98, *p* = 0.005) and 5-year OS (RR: 0.93, 95% CI: 0.88–0.98, *p* = 0.007) in the NCO group were statistically significantly higher than in CO group. For both outcomes, the detected heterogeneity was low but statistically negligible (3-year OS–I^2^ = 0%, *p* = 0.72; 5-year OS–I^2^ = 5%, *p* = 0.38).

#### 3.3.2. Disease-Free Survival

In total, five out of eight included studies [16,17,19,20,21] comprising 1458 patients (CO 736, NCO 722) reported both 3-year and 5-year DFSs (see Supplementary Materials, Figures S1 and S2). Meta-analysis of the pooled results showed a non-statistically significant difference between the two groups as concerned both outcomes (3-year DFS—RR: 0.97, 95% CI: 0.90–1.04, *p* = 0.44; 5-year DFS—RR: 0.98, 95% CI: 0.90–1.06, *p* = 0.60). For both outcomes, detected heterogeneity was moderate but statistically negligible (3-year DFS—I^2^ = 36%, *p* = 0.18; 5-year DFS—I^2^ = 40%, *p* = 0.16).

#### 3.3.3. Recurrences

In total, six of the eight included studies [16,17,19,20,21,30] comprising 1880 patients (CO 910, NCO 970) reported an overall recurrence rate (see Supplementary Materials, Figure S3). Meta-analysis of pooled results showed a non-statistically significant difference between the two groups (RR: 1.13, 95% CI: 0.94–1.35, *p* = 0.19). Heterogeneity was low but statistically negligible (I^2^ = 10%, *p* = 0.35).

Moreover, five of the eight included studies [16,19,20,21,30] comprising 1734 patients (CO 830, NCO 904) reported recurrence rates in the peritoneum (see Supplementary Materials, Figure S4). Meta-analysis of the pooled results showed a non-statistically significant difference between the two groups (RR: 1.11, 95% CI: 0.85–1.44, *p* = 0.43). Heterogeneity was low but statistically non-significant (I^2^ = 0%, *p* = 0.83).

Further, five of the eight included studies [16,19,20,21,30] comprising 1734 patients (CO 830, NCO 904) reported recurrence rates in other sites (see Supplementary Materials, Figure S5). Meta-analysis of the pooled results showed a non-statistically significant difference between the two groups (RR: 1.02, 95% CI: 0.79–1.31, *p* = 0.88). Heterogeneity was low but statistically negligible (I^2^ = 0%, *p* = 0.88).

#### 3.3.4. Operative Time

**Figure 4:**
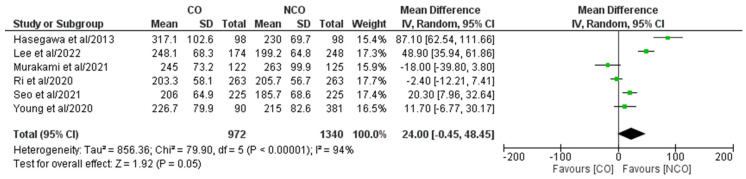
Forest plot comparing operative time between the CO and NCO groups. CI, confidence interval; M–H, Mantel–Haenszel [16,18,19,21,27,30].

In total, six out of eight included studies [16,18,19,21,27,30] comprising 2312 patients (CO 972, NCO 1340) reported operative time (Figure 4). Meta-analysis of the pooled results showed a statistically significant longer operative time for the CO group compared to the NCO group (MD: 24.00, 95% CI: −0.45–48.45, *p* = 0.05). Heterogeneity was high and statistically significant (I^2^ = 94%, *p* < 0.00001).

#### 3.3.5. Estimated Blood Loss

**Figure 5:**
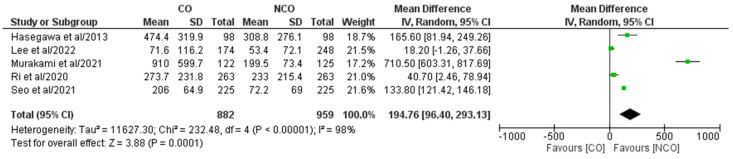
Forest plot comparing estimated blood loss between the CO and NCO groups. CI, confidence interval; M–H, Mantel–Haenszel [16,19,21,27,30].

In total, five of the eight included studies [16,19,21,27,30] comprising 1841 patients (CO 882, NCO 959) reported estimated blood loss (Figure 5). Meta-analysis of the pooled results showed a statistically significant higher estimated blood loss for the CO group, when compared to the NCO group (MD: 194.76, 95% CI: 96.40–293.13, *p* = 0.0001). Heterogeneity was high and statistically significant (I^2^ = 98%, *p* < 0.00001).

#### 3.3.6. Number of Lymph Nodes Harvested

In total, six of the eight included studies [17,18,19,21,27,30] comprising 2262 patients (CO 954, NCO 1308) reported harvested lymph nodes number (see Supplementary Materials, Figure S6). Meta-analysis of the pooled results showed a non-statistically significant difference between the two groups (MD: −0.52, 95% CI: −3.44–2.40, *p* = 0.73). Heterogeneity was high and statistically significant (I^2^ = 76%, *p* = 0.0009).

#### 3.3.7. Overall Complications

In total, six of the eight included studies [16,18,19,20,21,30] comprising 2205 patients (CO 920, NCO 1285) reported an overall complication rate (see Supplementary Materials, Figure S7). Meta-analysis of the pooled results showed a statistically non-significant difference between the two groups (RR: 1.20, 95% CI: 0.93–1.54, *p* = 0.17). Heterogeneity was high and statistically relevant (I^2^ = 55%, *p* = 0.05).

#### 3.3.8. Major Complications

In total, five of the eight included studies [19,20,21,27,30] comprising 1785 patients (CO 854, NCO 931) reported rates of major complications (see Supplementary Materials, Figure S8). Meta-analysis of the pooled results showed a statistically non-significant difference between the two groups (RR: 0.88, 95% CI: 0.51–1.52, *p* = 0.65). Heterogeneity was high and statistically relevant (I^2^ = 58%, *p* = 0.05).

#### 3.3.9. Subgroup Analysis

Subgroup analysis was carried out according to discrepancies in study designs. In particular, we analyzed the different outcomes just considering PSM studies. Our subgroup analysis confirmed the outcomes of pooled analysis: higher 3-year OS and 5-year OS rates in the NCO group, longer operative time and higher estimated blood loss in the CO group (see Supplementary Materials, Figures S9–S15).

#### 3.3.10. Publication Bias

According to the Cochrane Handbook for Systematic Reviews of Interventions (Version 5.1.0), tests for funnel plot asymmetry should only be carried out when meta-analysis includes at least 10 studies, as a smaller number of studies reduces the potential of tests to identify the case from real asymmetry [35]. As our meta-analysis included eight studies, we did not perform an analysis of publication bias.

## 4. Discussion

Gastrectomy with D2 lymphadenectomy represents a cornerstone in curative treatment for AGC [36]. However, despite correct radical intent gastrectomy, GC was marked by a 40–61% recurrence rate [37]. Peritoneal recurrence stood out, representing 36–45% of all kinds [38]. Omentum turned out as the most involved peritoneal site [6,7,14,22,23,24,25,26].

Omentum is a tissue stemming from the dorsal mesogastrium around the eighth week of gestation [15,39]. It consists of two mesothelial layers, mainly enclosing adipocytes embedded in a loose connective tissue and accumulations of mononuclear phagocytic cells [15,40]. Omentum has a rich vascularity with many widespread typical convolutions termed omental glomeruli, because they resemble renal glomeruli [15,40]. Omentum’s leukocytes gather in the perivascular area and form what we call “milky spots” [15,41]. Cells are arranged around omental glomeruli, which lie directly under the mesothelium [15,42,43]. They are supported by frail networks of reticular fibers that make up the organ’s structure [15,44]. In humans, milky spots include macrophages (70%), B-lymphocytes (10%), T-lymphocytes (10%), mast cells and stromal cells [15,45]. Macrophages in mature omentum are mainly scavengers [15,46]. They seem to be different from monocytic precursors in milky spots and are not dependent on bone-marrow-derived precursors [15,46]. They are dendritic in shape and have remarkable phagocytic abilities [15,46]. When activated, macrophage precursors in milky spots proliferate, then migrate to the mesothelial surface and turn into dendritic-shaped macrophages [15,46].

Omentum has turned out as a frequent site of metastatic malignancy [15,47,48]. In animals, tumor cells that are inoculated into the peritoneal cavity preferentially infiltrate omentum milky spots and grow into distinct metastases [15,47,48]. Omentum seems to be able to support not only malignant cells in milky spots, but also free intraperitoneal cells [15,47,48]. This happens thanks to omentum intrinsic angiogenic properties, as recent studies have highlighted [15,47,48,49]. In animals, omentum removal affects the survival of free intraperitoneal malignant cells and consequently reduces the local recurrence rate [15,50,51]. In this background, the omentum is often removed as part of resected malignancies in various intra-abdominal organs (ovarian cancer and GC, in particular) [15].

Despite omental tissue’s protumoral properties, authors are still debating on the need to perform CO, in addition to gastrectomy and D2 lymphadenectomy for GC [6,7,14,22,23,24,25,26]. Analyses of major guidelines in GC treatment clearly explained this topic. Japanese gastric cancer treatment guidelines 2018 (5th edition) suggested total removal of greater omentum in patients affected by T3 gastric cancer or deeper tumors, as an integral part of radical gastrectomy [52]. For patients affected by T1-T2 gastric cancer, the omentum more than 3 cm away from the gastroepiploic artery may be preserved [52]. Recommendations by the Japanese Gastric Cancer Association (JGCA) were shared by the Italian Research Group for Gastric Cancer (GIRCG) [53]. Following the aforementioned Japanese guidelines, the Chinese Society of Clinical Oncology (CSCO) stressed the important role of omentectomy by recommending total omentum removal in both D1 and D2 gastrectomy [54]. No mention of the role of omentectomy in radical gastrectomy was made by the Korean Practice Guideline for Gastric Cancer 2018 and National Comprehensive Cancer Network^®^ (NCCN^®^) Guidelines for Gastric Cancer, Version 2.2022 [55].

In 2016, Jongerius et al. published a prospective multi-center cohort study (OMEGA trial), whose aim was to evaluate both incidence and risk factors for metastasis in the greater omentum, as regarded patients undergoing gastrectomy and CO for GC [56]. In a 100-patient population, the authors identified a low rate of incidence for omental metastases (5%) [56]. In addition, multivariate analysis identified that omental metastases were significantly related to microscopically non-radical resection, tumor expansion in the esophagus or duodenum, linitis plastica, tumor location in the proximal third of stomach, ≥5 cm tumor, III-IV stage of disease and category (y) pM1 [56].

Our meta-analysis aimed at updating data related to the comparison between patients who underwent gastrectomy with CO or without CO for AGC. We identified eight studies: seven retrospective ones and just one RCT [16,17,18,19,20,21,27,30]. Six out of seven retrospective studies had been developed as PSM [16,17,19,20,21,30]. Our meta-analysis indicated gastrectomy without CO as significantly associated with longer 3- and 5-year OSs, in the absence of relevant differences in terms of 3- and 5-year DFSs, overall recurrence, recurrences in peritoneum and recurrences in other site rates. Moreover, taking into account short-term outcomes, we found longer operative time and higher estimated blood loss in the CO group compared to the NCO group, with no statistically relevant differences between the two groups, as concerned the number of harvested lymph nodes, overall complications and major ones. However, it is necessary to consider that the significant differences identified between the surgical outcomes of the meta-analyzed studies (overall/major complications, operative time and estimated blood loss, in particular) could be influenced by different surgical procedures adopted. In particular, in some of the included studies it is possible to identify mixed populations of patients who underwent partial or complete omentectomy alone, as part of the bursectomy [16], or associated with splenectomy [16,17,19,20,27], transverse colectomy [17,20,27], cholecystectomy [19,27], partial pancreatectomy [20], adrenalectomy [27] and thoracotomy [27]. The above results were then confirmed by study design-based (PSM) subgroup analysis and seemed to be in line with reports in previous meta-analyses.

To date, just one RCT has been published comparing outcomes between CO and NCO gastrectomies for AGC [27]. Murakami et al. randomized a 251 overall patient population (CO: 125 patients, NCO: 126 patients) [27]. Following the exclusion of patients with peritoneal metastases or a history of laparotomy, 247 patients’ data were analyzed [27]. CO group showed a significantly longer median operative time (225 min vs. 204 min, *p* = 0.022) and was inclined to have higher median blood loss (260 mL vs. 210 mL, *p* = 0.073) [26]. Incidence of morbidity showed similar values, by recording a 10% rate in both groups (8% vs. 9%, *p* = 1000) [27]. No mortality was recorded in either group, although authors did not analyze survival outcomes [27].

As stated at the beginning of our discussion, surgery represents a cornerstone in the treatment of GC as well as a method that can lead to a higher chance of recovery [36,57]. Nevertheless, surgery is not always sufficient and feasible for patients affected by AGC [57]. A multimodal method that includes systemic or local therapies (neoadjuvants or adjuvants) may lead to greater disease control, ease of complete resection, and above all, improved survival outcomes [57]. Present multimodal strategies reflect significant geographical differences [58,59]. Adjuvant chemotherapy is the preferred treatment in East Asia, while neoadjuvant chemotherapy and adjuvant chemoradiation are the preferred ones in Europe and North America, respectively [58,59].

In particular, the application or non-application of neoadjuvant chemotherapy with or without radiotherapy could play a paramount role in highlighting differences between OS and DFS among groups to be compared. Different European trials that focused on the potential role of neoadjuvant chemotherapy collected interesting results [58,59]. At the end of the UK Medical Research Council Adjuvant Gastric Infusional Chemotherapy (MAGIC) trial, which included 503 resectable GC patients randomly assigned to three cycles of neoadjuvant chemotherapy (epirubicin, cisplatic and 5-fluorouracil) and surgery alone, neoadjuvant group showed significantly better 5-year OS (36% versus 23%; HR 0.75, *p* = 0.009) and DFS (35% versus 25%; HR 0.66, *p* < 0.001) [60]. The later Federation Nationale des Centres de Lutte contre le Cancer (FNCLCC)/the Federation Francophone de Cancerologie Digestive (FFCD) trial, that employed 5-fluorouracil plus cisplatin in preoperative setting and whose 224 resectable GC patients were randomly assigned to neoadjuvant chemotherapy and surgery alone, showed significantly improved R0 resection rates (87% versus 74%; *p* = 0.04), in addition to 5-year OS (38% versus 24%; HR 0.69, *p* = 0.02) and DFS (34% versus 19%; HR 0.65, *p* = 0.003) [61]. Similar outcomes were achieved by European Organisation for Research and Treatment of Cancer (EORTC) 40954 trial, whose 144 AGC patients were randomized to neoadjuvant 5-fluorouracil plus cisplatin or surgery alone [62]. Despite early termination, due to poor enrolment, the study highlighted a significant improvement in R0 resection rates for neoadjuvant group (81.9% versus 66.7%; *p* = 0.036) [62].

Two main Asian trials led to the most recent results. Korean PRODIGY trial compared neoadjuvant docetaxel, oxaliplatin and S-, followed by surgery and adjuvant S-1 with surgery and adjuvant S-1 (266 patients with resectable AGC) [63]. The study found a significant dominance of neoadjuvant chemotherapy in terms of 3-year DFS (66.3% versus 60.2%; HR 0.70, *p* = 0.023) [63]. Similar results were obtained from the Chinese RESOLVE trial, whose 1022 resectable AGC patients were randomized to neoadjuvant S-1 and oxaliplatin or adjuvant oxaliplatin or capecitabine/oxaliplatin, thus recording significantly improved 3-year DFS in the neoadjuvant group (62% versus 54.8%; HR 0.79, *p* = 0.045) [64].

With the exception of the Young et al. study [18], whose related data to neoadjuvant chemotherapy administration are unavailable, all studies included in our meta-analysis stem from Asian countries. This could be the reason why just a few patients undergoing neoadjuvant chemotherapy can be identified among the populations under analysis (Hasegawa et al. NCO 2/98 CO 6/98 [16]; Kim et al. n/a [17]; Ri et al. NCO 0/263 CO 0/263 [19]; Sakimura et al. NCO 5/70 CO 7/70 [20]; Seo et al. NCO 0/225 CO 0/225 [21]; Murakami et al. NCO 0/122 CO 0/125 [27]; Lee et al. NCO 0/174 CO 0/248 [30]). Therefore, we cannot fully rule out that the administration of neoadjuvant chemotherapy modifies long-term oncological outcomes in a more or less significant way, going so far as to nullify discrepancies between NCO and CO surgical groups.

To date, the development of further, possibly multi-center, randomized controlled trials corroborating the beneficial role of neoadjuvant chemotherapy in AGC patients is strongly needed.

### Limitations

Our research bears some limitations: (i) the literature search did not include non-English written scientific articles; (ii) included studies were almost exclusively retrospective or retrospective PSM ones; (iii) data related to original baseline patient were missing, although matchings were conducted in PSM studies; (iv) most studies stemmed from Asian countries; (v) size of analyzed populations was small; (vi) significant heterogeneities existed among three short-term outcomes, which might have an adverse impact on evidence for short-term outcomes. For all these reasons, a direct comparison of results turned out as difficult.

## 5. Conclusions

Gastrectomy with D2 lymphadenectomy is the gold standard in AGC treatment, although peritoneal recurrence rate is both non-negligible and a major cause of poor prognosis. Omentum is one of the most common metastatic sites.

Many Authors describe CO as an integral part of AGC surgical treatment, although CO role is still highly debated. In fact, no agreement has been reached among international guidelines yet.

Our updated meta-analysis found that NCO group had a statistically greater rate in 3-year and 5-year Oss than the CO group, while the CO group had significantly longer operative time and higher estimated blood loss than the NCO group.

However, given non-negligible bias among the meta-analyzed studies, our results need an extremely careful data reading. Therefore, further randomized, possibly multi-center trials may turn out of paramount importance in confirming our results.

## Figures and Tables

**Table 1:** Study characteristics.

Authors/Year	Study Type	Study Country	Study Period	Group	Patient Population, n	Gender, n		Age (Years), Mean or Median	BMI (kg/m^2^), Mean or Median
						Male	Female		
Lee et al./2022 [30]	Retrospective PSM	Korea	2014–2018	CO	174	122	52	59.9 ± 12.7	23.1 ± 3.7
				NCO	248	177	71	61.6 ± 13.3	23.8 ± 3.1
Seo et al./2021 [21]	Retrospective PSM	Korea	2003–2015	CO	225	131	94	59 (49–70)	23.5 (21.1–25.6)
				NCO	225	137	88	56 (49–67)	22.9 (21.0–24.9)
Murakami et al./2021 [27]	RCT	Japan	2011–2018	CO	122	89	33	71 (30–90)	22.4 (14.8–31.8)
				NCO	125	89	36	74 (45–89)	22.2 (14.5–32.1)
Sakimura et al./2020 [20]	Retrospective PSM	Japan	2008–2017	CO	70	46	24	65.0 (37–90)	22.2 (15.8–30.3)
				NCO	70	48	22	66.5 (42–94)	22.4 (16.4–32.6)
Ri et al./2020 [19]	Retrospective PSM	Japan	2006–2012	CO	263	176	87	66.7 ± 11	22.4 ± 3.6
				NCO	263	181	82	65.7 ± 12.9	22.5 ± 3.4
Young et al./2020 [18]	Retrospective	USA	2008–2016	CO	90	62	28	69.5 (62–77)	27.4 ± 6.1
				NCO	381	217	164	68 (58–76)	26.2 ± 5.3
Kim et al./2014 [17]	Retrospective PSM	Korea	2004–2011	CO	80	56	24	60.9 ± 11.2	n/a
				NCO	66	50	16	62.2 ± 11	n/a
Hasegawa et al./2013 [16]	Retrospective PSM	Japan	2000–2009	CO	98	72	26	69.0 (40–91)	n/a
				NCO	98	72	26	68.7 (45–91)	n/a

n = number; BMI = Body Mass Index; PSM = propensity score matching; RCT = randomized controlled trial; CO = complete omentectomy; NCO = non-complete omentectomy; n/a = not available [16,17,18,19,20,21,27,30].

**Table 2:** General population characteristics.

Authors/Year	Group	Patient Population, n	Surgical Approach, n		Surgical Procedure, n		Lymphadenectomy, n				pT Stage, n					pN Stage, n				pTNM Stage, n			
			Open	MIS	DG	TG	D1	D1+	D2	D2+	T0	T1	T2	T3	T4	N0	N1	N2	N3	I	II	III	IV
Lee et al./2022 [30]	CO	174	0	174	101	73	n/a	n/a	n/a	n/a	0	0	22	78	74	42	19	39	74	10	43	121	0
	NCO	248	0	248	157	91	n/a	n/a	n/a	n/a	0	0	45	119	84	65	35	62	86	21	77	150	0
Seo et al./2021 [21]	CO	225	60	165	167	58	0	25	200	0	0	0	0	100	125	75	42	42	66	0	95	130	0
	NCO	225	69	156	169	56	0	22	203	0	0	0	0	111	114	73	47	42	63	0	99	126	0
Murakami et al./2021 [27]	CO	122	122	0	73	49	0	0	122	0	0	20	21	42	39	44	29	25	24	26	48	41	7
	NCO	125	125	0	81	44	0	0	125	0	0	31	21	31	42	54	25	17	29	38	40	40	7
Sakimura et al./2020 [20]	CO	70	41	29	45	25	0	9	61		1	5	12	32	20	22	14	14	20	n/a	n/a	n/a	n/a
	NCO	70	25	45	44	26	0	14	56		0	6	16	27	21	29	14	9	18	n/a	n/a	n/a	n/a
Ri et al./2020 [19]	CO	263	263	0	156	107	11	146	106		0	47		216		148	0	115	0	28	101	129	5
	NCO	263	263	0	151	112	8	146	109		0	48		215		145	0	118	0	29	96	131	7
Young et al./2020 [18]	CO	90	n/a	n/a	n/a	n/a	n/a	n/a	n/a	n/a	n/a	n/a	n/a	41		47	53			n/a	n/a	n/a	n/a
	NCO	381	n/a	n/a	n/a	n/a	n/a	n/a	n/a	n/a	n/a	n/a	n/a	184		176	205			n/a	n/a	n/a	n/a
Kim et al./2014 [17]	CO	80	0	80	61	19	0	2	78	0	0	0	28	52	0	40	14	13	13	17	39	24	0
	NCO	66	0	66	54	12	0	5	61	0	0	0	37	29	0	34	8	16	8	23	26	17	0
Hasegawa et al./2013 [16]	CO	98	98	0	52	46	0	12	86	0	0	0	30	34	34	39	25	18	16	16	40	42	0
	NCO	98	84	14	61	37	0	13	85	0	0	0	34	30	34	41	23	17	17	21	36	41	0

n = number; CO = complete omentectomy; NCO = non-complete omentectomy; MIS = minimally invasive surgery; DG = Distal gastrectomy; TG = Total gastrectomy; n/a = not available [16,17,18,19,20,21,27,30].

## Data Availability

The data presented in this study are available on request from the corresponding author.

## Supplementary Materials

The following supporting information can be downloaded at: https://www.mdpi.com/article/10.3390/medicina58091241/s1, Table S1. Retrospective studies and retrospective PSMs evaluated using ROBINS-I; Table S2. Randomized controlled trial evaluated using RoB 2.0; Figure S1. Forest plot comparing 3-year DFS between the CO and NCO groups. CI, confidence interval; M–H, Mantel–Haenszel; Figure S2. Forest plot comparing 5-year DFS between the CO and NCO groups. CI, confidence interval; M–H, Mantel–Haenszel; Figure S3. Forest plot comparing overall recurrences between the CO and NCO groups. CI, confidence interval; M–H, Mantel–Haenszel; Figure S4. Forest plot comparing recurrence in peritoneum between the CO and NCO groups. CI, confidence interval; M–H, Mantel–Haenszel; Figure S5. Forest plot comparing recurrences in other sites between the CO and NCO groups. CI, confidence interval; M–H, Mantel–Haenszel; Figure S6. Forest plot comparing number of harvested lymph nodes between the CO and NCO groups. CI, confidence interval; M–H, Mantel–Haenszel; Figure S7. Forest plot comparing overall complications between the CO and NCO groups. CI, confidence interval; M–H, Mantel–Haenszel; Figure S8. Forest plot comparing major complications between the CO and NCO groups. CI, confidence interval; M–H, Mantel–Haenszel; Figure S9. Forest plot comparing 3-year OS between the CO and NCO PSM groups. CI, confidence interval; M–H, Mantel–Haenszel; Figure S10. Forest plot comparing 5-year OS between the CO and NCO PSM groups. CI, confidence interval; M–H, Mantel–Haenszel; Figure S11. Forest plot comparing operative time between the CO and NCO PSM groups. CI, confidence interval; M–H, Mantel–Haenszel; Figure S12. Forest plot comparing estimated blood loss between the CO and NCO PSM groups. CI, confidence interval; M–H, Mantel–Haenszel; Figure S13. Forest plot comparing number of harvested lymph nodes between the CO and NCO PSM groups. CI, confidence interval; M–H, Mantel–Haenszel; Figure S14. Forest plot comparing overall complications between the CO and NCO PSM groups. CI, confidence interval; M–H, Mantel–Haenszel; Figure S15. Forest plot comparing major complications between the CO and NCO PSM groups. CI, confidence interval; M–H, Mantel–Haenszel.
