# Supplementary material for: Gastrectomy with or without Complete Omentectomy for Advanced Gastric Cancer: A Meta-Analysis
Source: Medicina (Kaunas). 2022 Sep 7;58(9):1241. doi: 10.3390/medicina58091241 (PMC9503724; doi:10.3390/medicina58091241)
Supplement: Supplementary file 1 [file medicina-58-01241-s001.zip › medicina-1856091-supplementary.pdf]

Supplementary material

Tables S1. Retrospective studies and retrospective PSMs evaluated using ROBINS-I.

		Hasegawa [2013]	Kim [2014]	Young [2020]	Ri [2020]	Sakimura [2020]	Seo [2021]	Lee [2022]
Preintervention	Confounding	Moderate[1]	Moderate[2]	Moderate[3]	Moderate[4]	Moderate[5]	Moderate[1]	Moderate[1]
	Selection bias	Low	Low	Low	Low	Low	Low	Low
Intraintervention	Classification of interventions	Low	Low	Serious[3]	Low	Low	Low	Low
Postintervention	Intended interventions	Low	Low	Low	Low	Low	Low	Low
	Missing data	Low	Low	Low	Low	Low	Low	Low
	Measurement of outcomes	Low	Low	Low	Low	Moderate[6]	Low	Low
	Reported results	Low	Low	Low	Low	Low	Low	Low
Overall bias		Moderate	Moderate	Serious	Moderate	Moderate	Moderate	Moderate

[1] Surgical techniques varied among patients including the use of minimal invasive surgery or total gastrectomy.
[2] Treatments were determined in accordance with patients' surgical indications and disease baseline.
[3] No detailed information regarding surgical intervention was reported.
[4] Surgical approaches differed among the institutions included.
[5] The eligibility for the treatment of patients was determined by the physician.
[6] Survival data were administered in terms of 3-year overall survival and 3-year relapse-free survival.

Tables S2. Randomized controlled trial evaluated using RoB 2.0.

	**Murakami [2021]**
Bias arising from the randomization process	Low risk
Bias owing to deviation from intended interventions	Low risk
Bias owing to missing outcome data	Low risk
Bias in measurement of the outcome	Low risk
Bias in selection of the reported result	Low risk
Overall risk of bias	Low risk

Study or Subgroup	CO Events	CO Total	NCO Events	NCO Total	Weight	Risk Ratio M-H, Random, 95% CI
Hasegawa et al/2013	71	98	75	98	14.4%	0.95 [0.80, 1.12]
Kim et al/2014	66	80	59	66	19.8%	0.92 [0.81, 1.05]
Ri et al/2020	203	263	203	263	29.2%	1.00 [0.91, 1.10]
Sakimura et al/2020	56	70	47	70	10.5%	1.19 [0.97, 1.46]
Seo et al/2021	164	225	179	225	26.1%	0.92 [0.83, 1.02]
**Total (95% CI)**		**736**		**722**	**100.0%**	**0.97 [0.90, 1.04]**
Total events	560		563			

Heterogeneity: Tau² = 0.00; Chi² = 6.26, df = 4 (P = 0.18); I² = 36%
Test for overall effect: Z = 0.77 (P = 0.44)

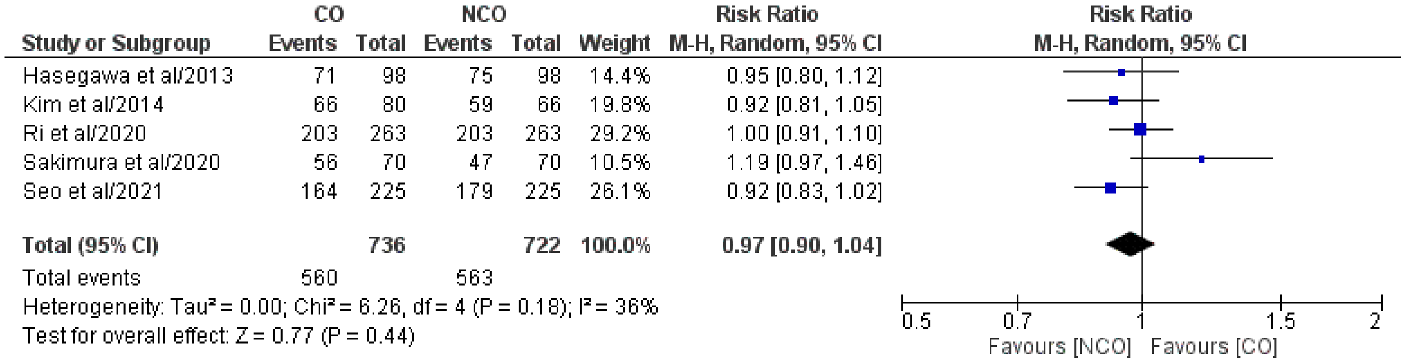

Figure S1. Forest plot comparing 3-year DFS between the CO and NCO groups. CI, confidence interval; M-H, Mantel–Haenszel. [16,17,19-21]

Study or Subgroup	CO Events	CO Total	NCO Events	NCO Total	Weight	Risk Ratio M-H, Random, 95% CI
Hasegawa et al/2013	65	98	66	98	12.4%	0.98 [0.81, 1.20]
Kim et al/2014	65	80	59	66	21.0%	0.91 [0.79, 1.04]
Ri et al/2020	194	263	195	263	28.3%	0.99 [0.90, 1.10]
Sakimura et al/2020	44	70	53	70	10.2%	0.83 [0.66, 1.04]
Seo et al/2021	179	225	166	225	28.1%	1.08 [0.97, 1.19]
**Total (95% CI)**		**736**		**722**	**100.0%**	**0.98 [0.90, 1.06]**
Total events	547		539			

Heterogeneity: Tau² = 0.00; Chi² = 6.64, df = 4 (P = 0.16); I² = 40%
Test for overall effect: Z = 0.52 (P = 0.60)

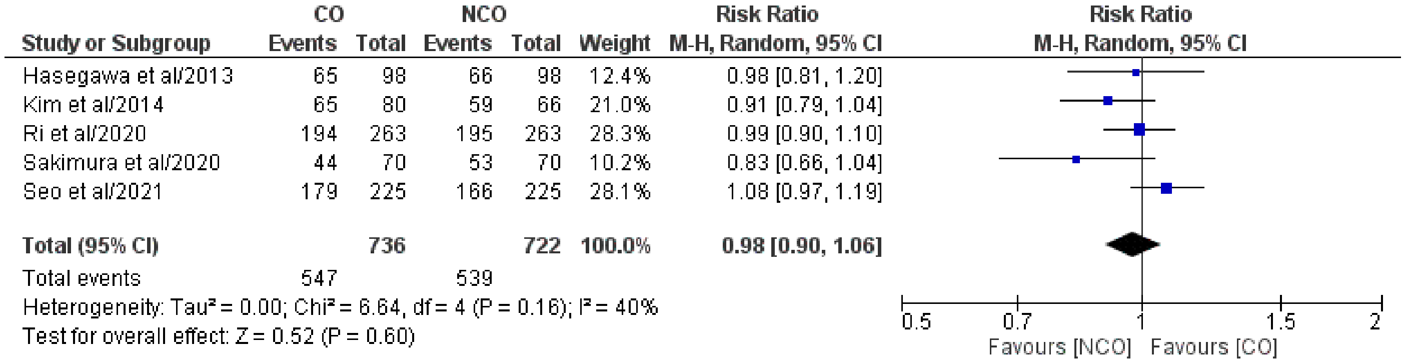

Figure S2. Forest plot comparing 5-year DFS between the CO and NCO groups. CI, confidence interval; M-H, Mantel–Haenszel. [16,17,19-21]

Study or Subgroup	CO Events	CO Total	NCO Events	NCO Total	Weight	Risk Ratio M-H, Random, 95% CI
Hasegawa et al/2013	18	98	20	98	9.1%	0.90 [0.51, 1.59]
Kim et al/2014	14	80	5	66	3.3%	2.31 [0.88, 6.08]
Lee et al/2022	35	174	51	248	18.9%	0.98 [0.67, 1.44]
Ri et al/2020	69	263	68	263	30.9%	1.01 [0.76, 1.35]
Sakimura et al/2020	24	70	15	70	9.7%	1.60 [0.92, 2.78]
Seo et al/2021	67	225	55	225	28.1%	1.22 [0.90, 1.65]
**Total (95% CI)**		**910**		**970**	**100.0%**	**1.13 [0.94, 1.35]**
Total events	227		214			

Heterogeneity: Tau² = 0.00; Chi² = 5.53, df = 5 (P = 0.35); I² = 10%
Test for overall effect: Z = 1.32 (P = 0.19)

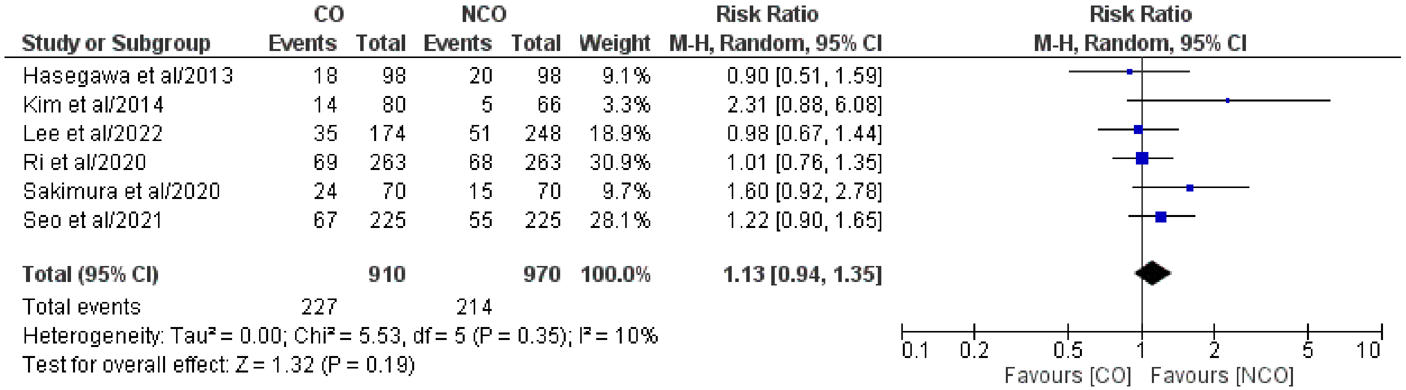

Figure S3. Forest plot comparing overall recurrences between the CO and NCO groups. CI, confidence interval; M-H, Mantel–Haenszel. [16,17,19-21,30]

	CO		NCO			Risk Ratio
**Study or Subgroup**	**Events**	**Total**	**Events**	**Total**	**Weight**	**M-H, Random, 95% CI**
Hasegawa et al/2013	7	98	7	98	6.8%	1.00 [0.36, 2.74]
Lee et al/2022	23	174	30	248	26.8%	1.09 [0.66, 1.82]
Ri et al/2020	28	263	29	263	28.7%	0.97 [0.59, 1.58]
Sakimura et al/2020	7	70	8	70	7.5%	0.88 [0.34, 2.28]
Seo et al/2021	35	225	25	225	30.1%	1.40 [0.87, 2.26]
**Total (95% CI)**		**830**		**904**	**100.0%**	**1.11 [0.85, 1.44]**
Total events	100		99			

Heterogeneity: Tau² = 0.00; Chi² = 1.49, df = 4 (P = 0.83); I² = 0%
Test for overall effect: Z = 0.78 (P = 0.43)

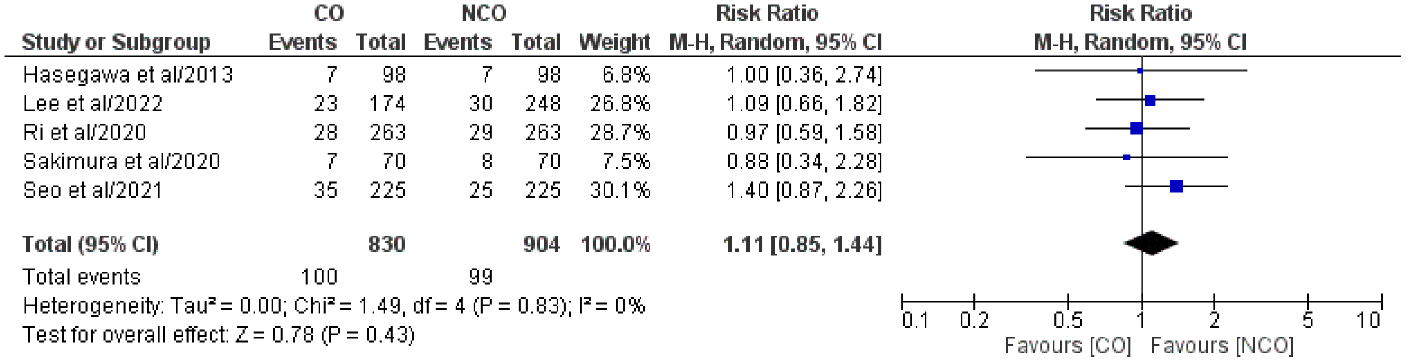

Figure S4. Forest plot comparing recurrence in peritoneum between the CO and NCO groups. CI, confidence interval; M-H, Mantel–Haenszel. [16,19-21,30]

	CO		NCO			Risk Ratio
**Study or Subgroup**	**Events**	**Total**	**Events**	**Total**	**Weight**	**M-H, Random, 95% CI**
Hasegawa et al/2013	11	98	13	98	11.0%	0.85 [0.40, 1.80]
Lee et al/2022	12	174	21	248	13.3%	0.81 [0.41, 1.61]
Ri et al/2020	41	263	39	263	38.1%	1.05 [0.70, 1.57]
Sakimura et al/2020	11	70	8	70	8.6%	1.38 [0.59, 3.21]
Seo et al/2021	32	225	30	225	29.0%	1.07 [0.67, 1.69]
**Total (95% CI)**		**830**		**904**	**100.0%**	**1.02 [0.79, 1.31]**
Total events	107		111			

Heterogeneity: Tau² = 0.00; Chi² = 1.19, df = 4 (P = 0.88); I² = 0%
Test for overall effect: Z = 0.15 (P = 0.88)

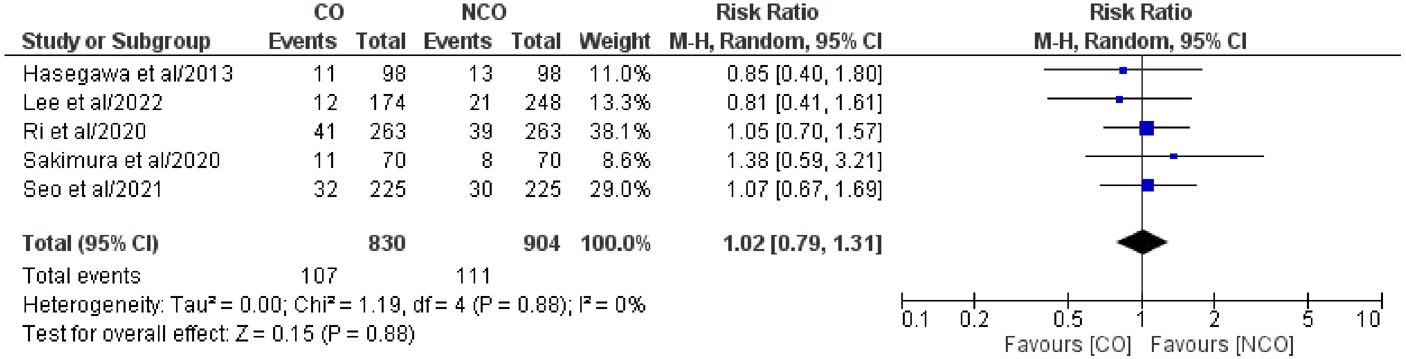

Figure S5. Forest plot comparing recurrences in other sites between the CO and NCO groups. CI, confidence interval; M-H, Mantel–Haenszel. [16,19-21,30]

Study or Subgroup	CO Mean	CO SD	CO Total	NCO Mean	NCO SD	NCO Total	Weight	Mean Difference IV, Random, 95% CI
Kim et al/2014	34.6	14.7	80	39.8	14.7	66	14.3%	-5.20 [-9.99, -0.41]
Lee et al/2022	73.6	23.8	174	69.3	23.5	248	14.8%	4.30 [-0.29, 8.89]
Murakami et al/2021	38.5	16.5	122	41.3	27.8	125	12.4%	-2.80 [-8.49, 2.89]
Ri et al/2020	41.3	14.2	263	39.7	14.9	263	20.0%	1.60 [-0.89, 4.09]
Seo et al/2021	44.7	17.9	225	42.7	16.4	225	18.3%	2.00 [-1.17, 5.17]
Young et al/2020	16	9.8	90	19.7	11.9	381	20.3%	-3.70 [-6.05, -1.35]
**Total (95% CI)**			**954**			**1308**	**100.0%**	**-0.52 [-3.44, 2.40]**

Heterogeneity: Tau² = 9.47; Chi² = 20.76, df = 5 (P = 0.0009); I² = 76%
Test for overall effect: Z = 0.35 (P = 0.73)

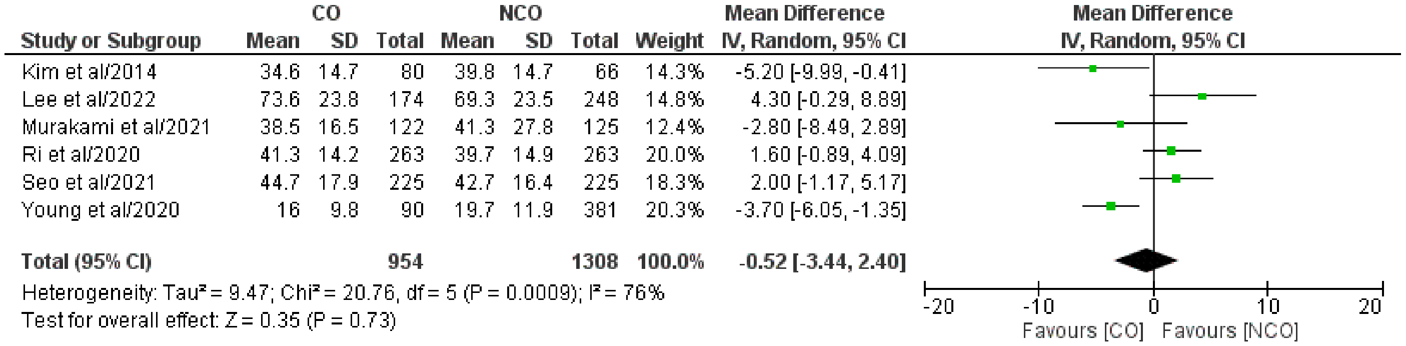

Figure S6. Forest plot comparing number of harvested lymph nodes between the CO and NCO groups. CI, confidence interval; M-H, Mantel–Haenszel. [17-19,21,27,30]

Study or Subgroup	CO Events	CO Total	NCO Events	NCO Total	Weight	Risk Ratio M-H, Random, 95% CI
Hasegawa et al/2013	40	98	37	98	20.3%	1.08 [0.76, 1.53]
Lee et al/2022	27	174	26	248	14.4%	1.48 [0.90, 2.45]
Ri et al/2020	72	263	42	263	20.7%	1.71 [1.22, 2.41]
Sakimura et al/2020	12	70	18	70	10.4%	0.67 [0.35, 1.28]
Seo et al/2021	17	225	11	225	8.8%	1.55 [0.74, 3.22]
Young et al/2020	43	90	182	381	25.4%	1.00 [0.79, 1.27]
**Total (95% CI)**		**920**		**1285**	**100.0%**	**1.20 [0.93, 1.54]**
Total events	211		316			

Heterogeneity: Tau² = 0.05; Chi² = 11.19, df = 5 (P = 0.05); I² = 55%
Test for overall effect: Z = 1.38 (P = 0.17)

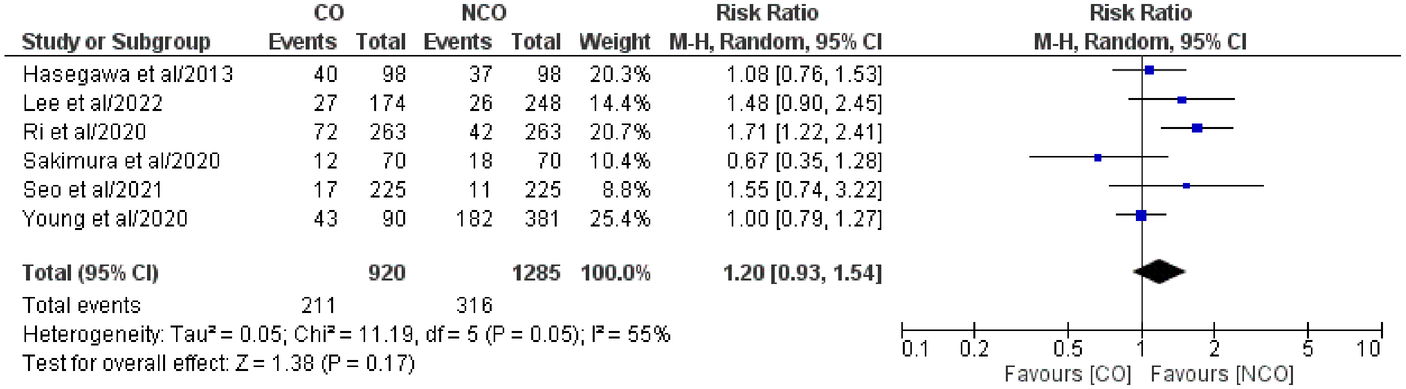

Figure S7. Forest plot comparing overall complications between the CO and NCO groups. CI, confidence interval; M-H, Mantel–Haenszel. [16,18-21,30]

Study or Subgroup	CO Events	CO Total	NCO Events	NCO Total	Weight	Risk Ratio M-H, Random, 95% CI
Lee et al/2022	6	174	11	248	16.7%	0.78 [0.29, 2.06]
Murakami et al/2021	10	122	11	125	19.8%	0.93 [0.41, 2.11]
Ri et al/2020	46	263	27	263	29.2%	1.70 [1.09, 2.65]
Sakimura et al/2020	4	70	8	70	13.7%	0.50 [0.16, 1.58]
Seo et al/2021	9	225	17	225	20.6%	0.53 [0.24, 1.16]
**Total (95% CI)**		**854**		**931**	**100.0%**	**0.88 [0.51, 1.52]**
Total events	75		74			

Heterogeneity: Tau² = 0.21; Chi² = 9.51, df = 4 (P = 0.05); I² = 58%
Test for overall effect: Z = 0.46 (P = 0.65)

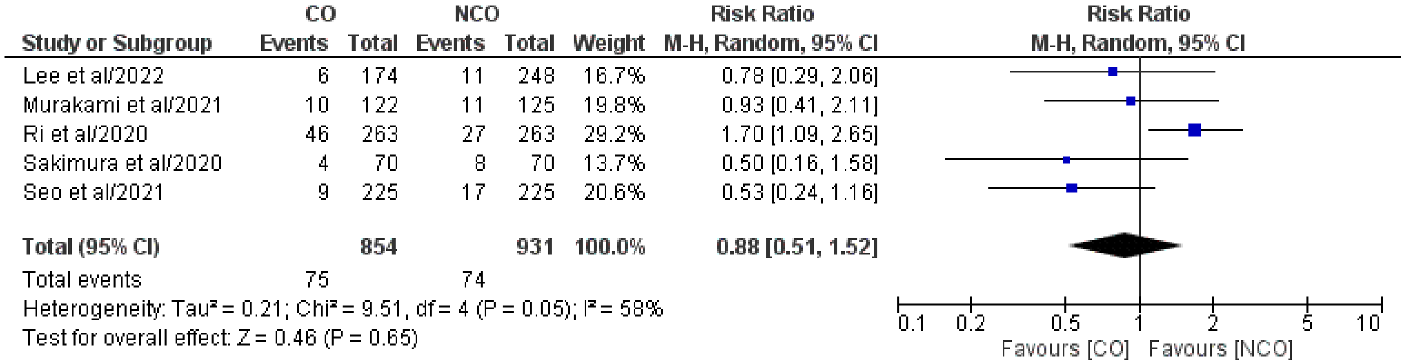

Figure S8. Forest plot comparing major complications between the CO and NCO groups. CI, confidence interval; M-H, Mantel–Haenszel. [19-21,27,30]

Study or Subgroup	CO Events	CO Total	NCO Events	NCO Total	Weight	Risk Ratio M-H, Random, 95% CI
Hasegawa et al/2013	76	98	88	98	12.1%	0.86 [0.76, 0.98]
Kim et al/2014	73	80	63	66	26.0%	0.96 [0.88, 1.04]
Ri et al/2020	216	263	222	263	32.6%	0.97 [0.90, 1.05]
Sakimura et al/2020	53	70	56	70	6.1%	0.95 [0.79, 1.13]
Seo et al/2021	174	225	189	225	23.2%	0.92 [0.84, 1.01]
**Total (95% CI)**		**736**		**722**	**100.0%**	**0.94 [0.90, 0.98]**
Total events	592		618			

Heterogeneity: Tau² = 0.00; Chi² = 2.90, df = 4 (P = 0.57); I² = 0%
Test for overall effect: Z = 2.73 (P = 0.006)

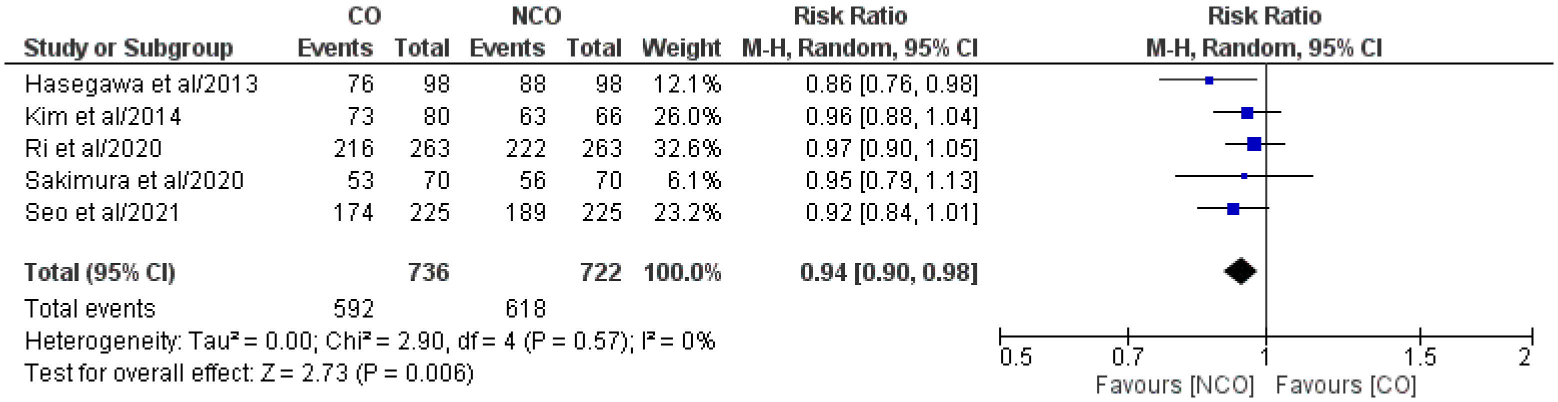

Figure S9. Forest plot comparing 3-year OS between the CO and NCO PSM groups. CI, confidence interval; M-H, Mantel–Haenszel. [16,17,19-21]

Study or Subgroup	CO Events	CO Total	NCO Events	NCO Total	Weight	Risk Ratio M-H, Random, 95% CI
Hasegawa et al/2013	65	98	78	98	11.0%	0.83 [0.70, 0.99]
Kim et al/2014	71	80	63	66	29.0%	0.93 [0.85, 1.02]
Ri et al/2020	203	263	209	263	30.8%	0.97 [0.89, 1.06]
Sakimura et al/2020	40	70	52	70	5.8%	0.77 [0.60, 0.98]
Seo et al/2021	163	225	170	225	23.3%	0.96 [0.86, 1.07]
**Total (95% CI)**		**736**		**722**	**100.0%**	**0.93 [0.87, 0.99]**
Total events	542		572			

Heterogeneity: Tau² = 0.00; Chi² = 5.10, df = 4 (P = 0.28); I² = 22%
Test for overall effect: Z = 2.41 (P = 0.02)

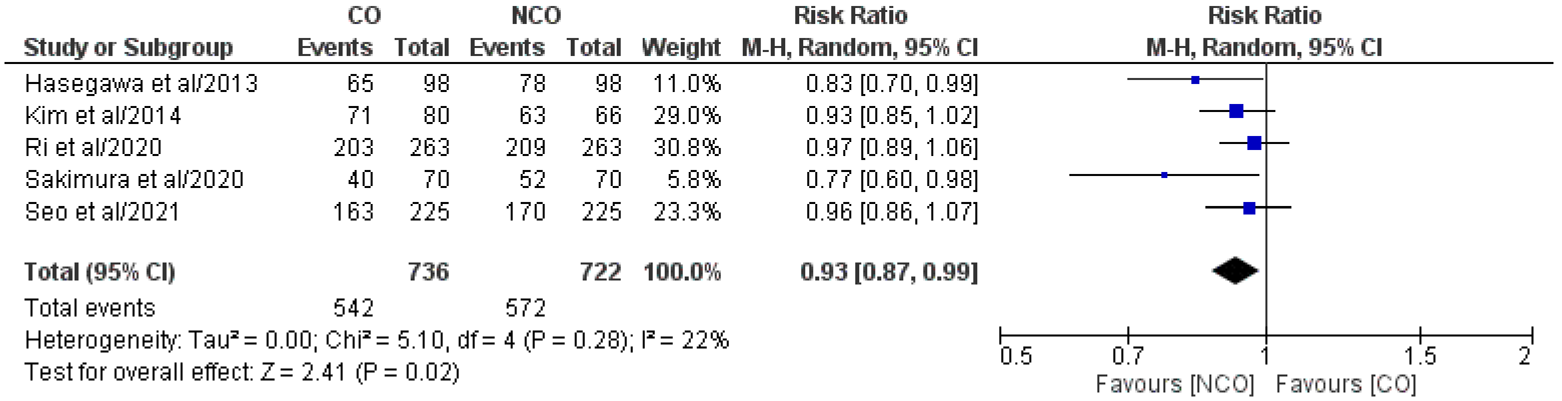

Figure S10. Forest plot comparing 5-year OS between the CO and NCO PSM groups. CI, confidence interval; M-H, Mantel–Haenszel. [16,17,19-21]

Study or Subgroup	CO Mean	CO SD	CO Total	NCO Mean	NCO SD	NCO Total	Weight	Mean Difference IV, Random, 95% CI
Hasegawa et al/2013	317.1	102.6	98	230	69.7	98	23.0%	87.10 [62.54, 111.66]
Lee et al/2022	248.1	68.3	174	199.2	64.8	248	25.5%	48.90 [35.94, 61.86]
Ri et al/2020	203.3	58.1	263	205.7	56.7	263	26.0%	-2.40 [-12.21, 7.41]
Seo et al/2021	206	64.9	225	185.7	68.6	225	25.6%	20.30 [7.96, 32.64]
**Total (95% CI)**			**760**			**834**	**100.0%**	**37.05 [5.18, 68.91]**

Heterogeneity: Tau² = 993.67; Chi² = 67.37, df = 3 (P < 0.00001); I² = 96%
Test for overall effect: Z = 2.28 (P = 0.02)

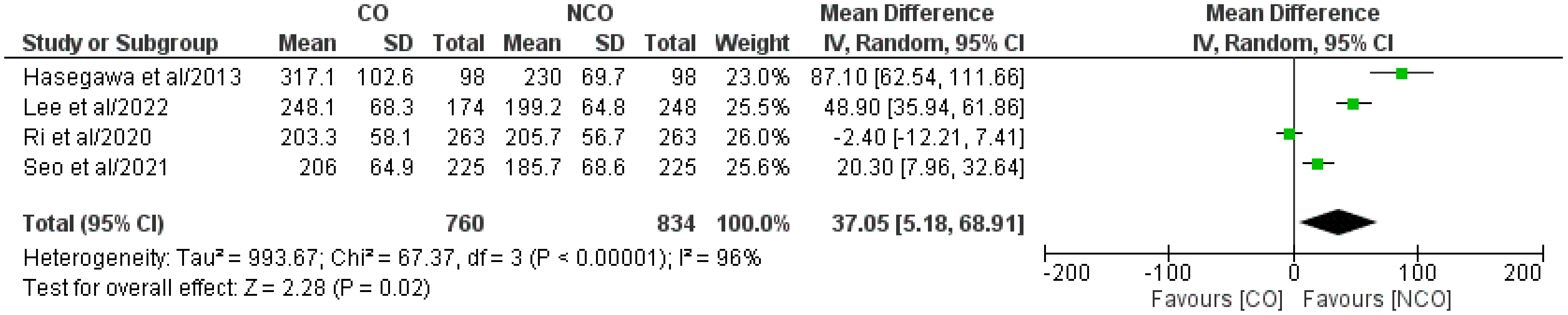

Figure S11. Forest plot comparing operative time between the CO and NCO PSM groups. CI, confidence interval; M-H, Mantel–Haenszel. [16,19,21,30]

Study or Subgroup	CO Mean	CO SD	CO Total	NCO Mean	NCO SD	NCO Total	Weight	Mean Difference IV, Random, 95% CI
Hasegawa et al/2013	474.4	319.9	98	308.8	276.1	98	20.5%	165.60 [81.94, 249.26]
Lee et al/2022	71.6	116.2	174	53.4	72.1	248	26.8%	18.20 [-1.26, 37.66]
Ri et al/2020	273.7	231.8	263	233	215.4	263	25.5%	40.70 [2.46, 78.94]
Seo et al/2021	206	64.9	225	72.2	69	225	27.1%	133.80 [121.42, 146.18]
**Total (95% CI)**			**760**			**834**	**100.0%**	**85.51 [9.71, 161.32]**

Heterogeneity: Tau² = 5475.12; Chi² = 107.86, df = 3 (P < 0.00001); I² = 97%
Test for overall effect: Z = 2.21 (P = 0.03)

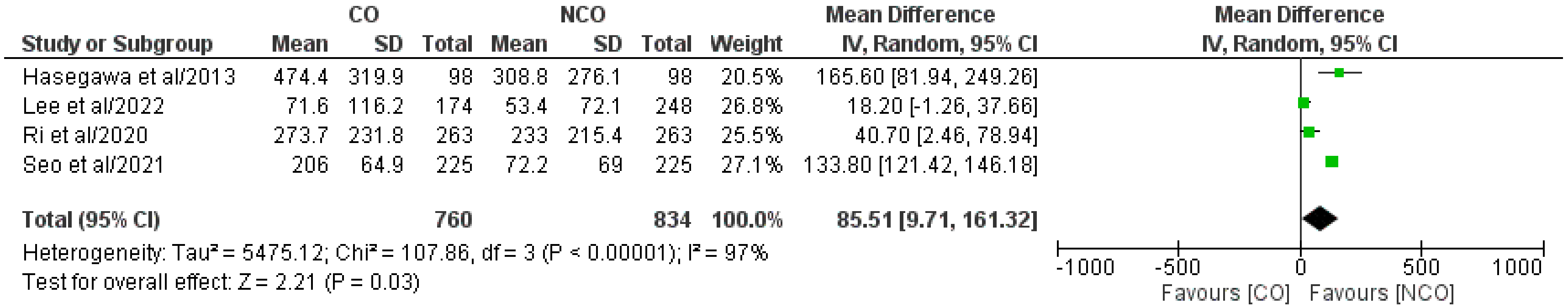

Figure S12. Forest plot comparing estimated blood loss between the CO and NCO PSM groups. CI, confidence interval; M-H, Mantel–Haenszel. [16,19,21,30]

	CO			NCO				Mean Difference
**Study or Subgroup**	**Mean**	**SD**	**Total**	**Mean**	**SD**	**Total**	**Weight**	**IV, Random, 95% CI**
Kim et al/2014	34.6	14.7	80	39.8	14.7	66	20.2%	-5.20 [-9.99, -0.41]
Lee et al/2022	73.6	23.8	174	69.3	23.5	248	21.0%	4.30 [-0.29, 8.89]
Ri et al/2020	41.3	14.2	263	39.7	14.9	263	31.1%	1.60 [-0.89, 4.09]
Seo et al/2021	44.7	17.9	225	42.7	16.4	225	27.6%	2.00 [-1.17, 5.17]
**Total (95% CI)**			**742**			**802**	**100.0%**	**0.90 [-2.21, 4.02]**

Heterogeneity: Tau² = 6.51; Chi² = 8.95, df = 3 (P = 0.03); I² = 66%
Test for overall effect: Z = 0.57 (P = 0.57)

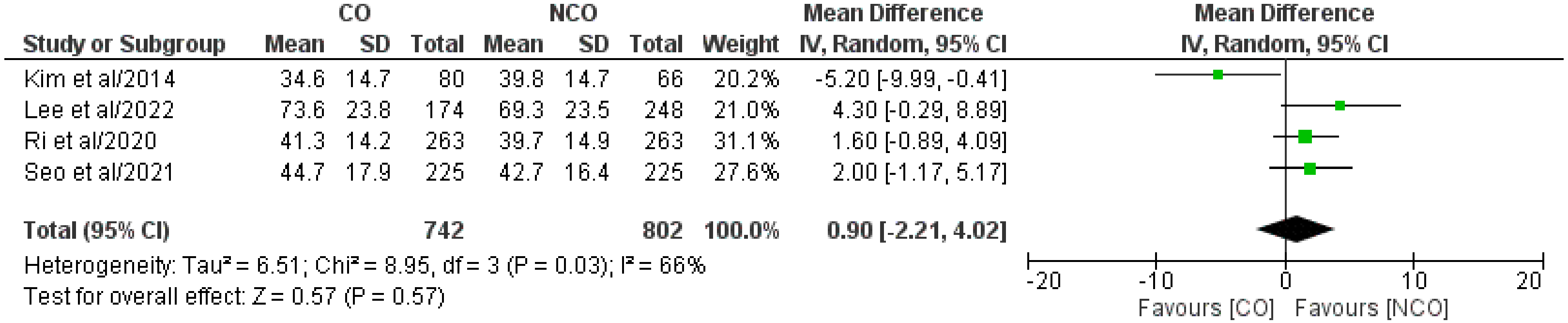

Figure S13. Forest plot comparing number of harvested lymph nodes between the CO and NCO PSM groups. CI, confidence interval; M-H, Mantel–Haenszel. [16,19,21,30]

	CO		NCO			Risk Ratio
**Study or Subgroup**	**Events**	**Total**	**Events**	**Total**	**Weight**	**M-H, Random, 95% CI**
Hasegawa et al/2013	40	98	37	98	26.8%	1.08 [0.76, 1.53]
Lee et al/2022	27	174	26	248	19.5%	1.48 [0.90, 2.45]
Ri et al/2020	72	263	42	263	27.2%	1.71 [1.22, 2.41]
Sakimura et al/2020	12	70	18	70	14.3%	0.67 [0.35, 1.28]
Seo et al/2021	17	225	11	225	12.2%	1.55 [0.74, 3.22]
**Total (95% CI)**		**830**		**904**	**100.0%**	**1.27 [0.94, 1.72]**
Total events	168		134			

Heterogeneity: Tau² = 0.06; Chi² = 8.17, df = 4 (P = 0.09); I² = 51%
Test for overall effect: Z = 1.53 (P = 0.13)

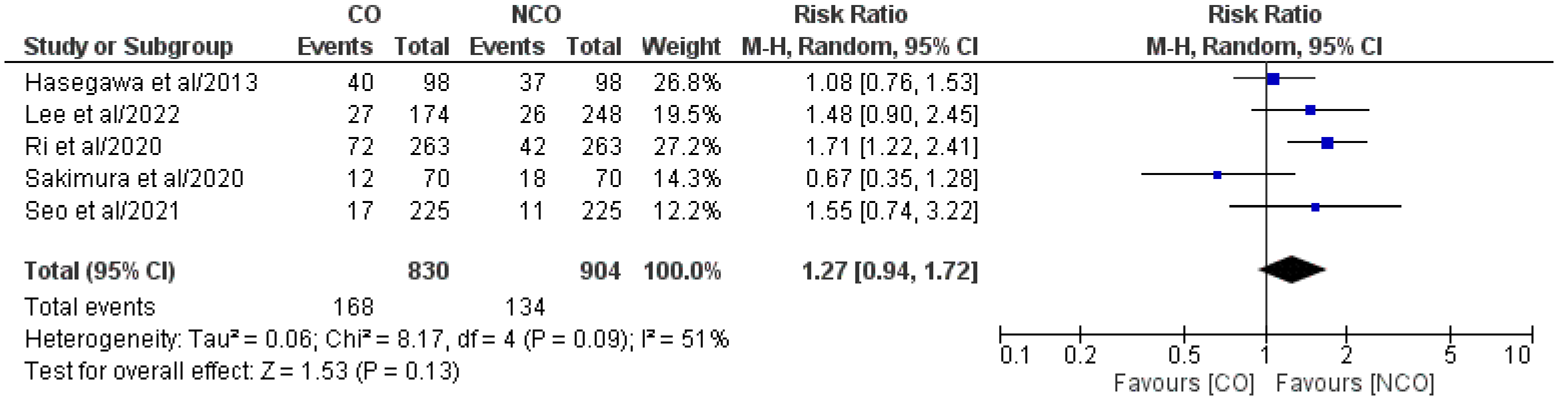

Figure S14. Forest plot comparing overall complications between the CO and NCO PSM groups. CI, confidence interval; M-H, Mantel–Haenszel. [16,19-21,30]

Study or Subgroup	CO Events	CO Total	NCO Events	NCO Total	Weight	Risk Ratio M-H, Random, 95% CI
Lee et al/2022	6	174	11	248	22.0%	0.78 [0.29, 2.06]
Ri et al/2020	46	263	27	263	33.3%	1.70 [1.09, 2.65]
Sakimura et al/2020	4	70	8	70	18.8%	0.50 [0.16, 1.58]
Seo et al/2021	9	225	17	225	25.9%	0.53 [0.24, 1.16]
**Total (95% CI)**		**732**		**806**	**100.0%**	**0.84 [0.42, 1.70]**
Total events	65		63			

Heterogeneity: Tau² = 0.33; Chi² = 9.36, df = 3 (P = 0.02); I² = 68%
Test for overall effect: Z = 0.48 (P = 0.63)

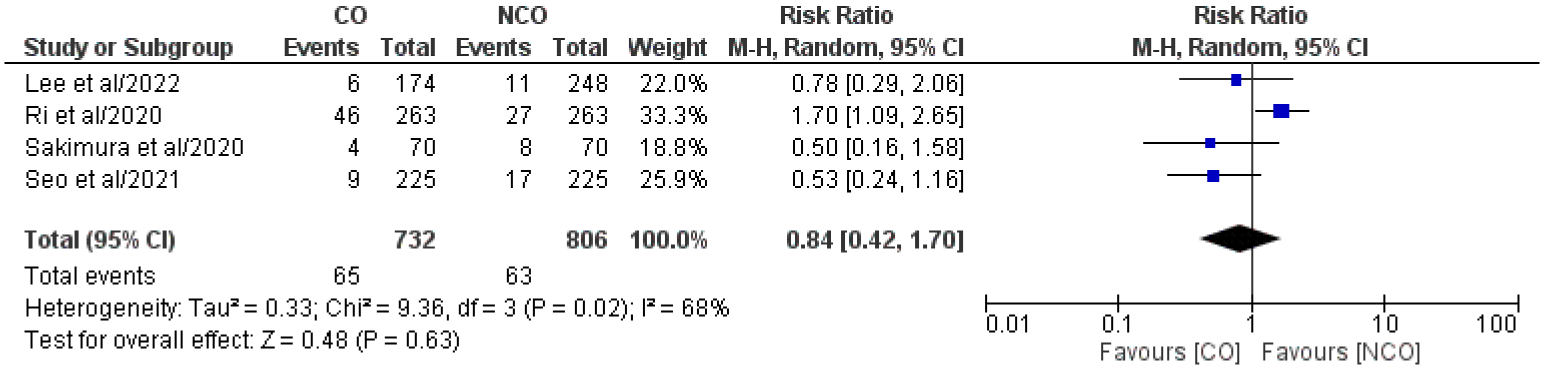

Figure S15. Forest plot comparing major complications between the CO and NCO PSM groups. CI, confidence interval; M-H, Mantel–Haenszel. [19-21,30]
